# A Novel Prognostic Nomogram for Patients With Recurrence of Intrahepatic Cholangiocarcinoma After Initial Surgery

**DOI:** 10.3389/fonc.2020.00434

**Published:** 2020-04-02

**Authors:** Kai-Li Xing, Liang-He Lu, Xin Huang, Chao-Bin He, Yun-Da Song, Rong-Ping Guo, Sheng-Ping Li

**Affiliations:** ^1^Department of Pancreatobiliary Surgery, Sun Yat-sen University Cancer Center, Guangzhou, China; ^2^State Key Laboratory of Oncology in South China, Guangzhou, China; ^3^Collaborative Innovation Center for Cancer Medicine, Sun Yat-sen University Cancer Center, Guangzhou, China; ^4^Department of Hepatobiliary Oncology of Sun Yat-sen University Cancer Center, Guangzhou, China

**Keywords:** intrahepatic cholangiocarcinoma, post-recurrence survival, resection, recurrence, nomogram

## Abstract

**Background:** The prognosis of patients with post-operative recurrent intrahepatic cholangiocarcinoma (ICC) is at great variance. We aimed to propose a novel efficient prognostic nomogram in facilitating the risk stratification for post-operative recurrent ICC patients.

**Methods:** From 2000 to 2016, a total of 237 post-operative recurrent ICC patients were enrolled in this study, and randomly divided into training (*n* = 178) and validation cohorts (*n* = 59) at a ratio of 3:1. The performance of this nomogram was assessed by discrimination, calibration, and clinical usefulness, and the results were compared with four other currently used ICC staging systems.

**Results:** On multivariate analysis of the training cohort, serum CA 19-9, albumin-bilirubin grade at recurrence, time from primary resection to recurrence, tumor number at recurrence, and treatment for recurrence were selected for the model. The concordance index (C-index) of our model was 0.791 [95% confidence interval (CI), 0.736–0.846], which was statistically higher than the values of the following systems: American Joint Committee on Cancer (AJCC) 8th edition (0.610), Liver Cancer Study Group of Japan (0.613), Nathan (0.582), and Okabayashi (0.600; *P* < 0.001 for all). The nomogram performed well in terms of calibration when compared with actual observation. The findings were supported by the validation cohort.

**Conclusions:** Compared with four currently used staging systems for ICC, our nomogram showed more promising clinical utility in improving individualized predictions of survival for post-operative recurrent ICC patients.

## Introduction

Intrahepatic cholangiocarcinoma (ICC) is the second most common primary liver cancer with increasing incidence and mortality worldwide (1). Surgical resection is the only potentially curative treatment for ICC (2, 3), but the prognosis remains far from homogenous, with the extremely high incidence of recurrence, which was reported to exceed 70% (4).

A wide variety of prognostic algorithms had been proposed for ICC patients over the years. The prognostic reliability and usability of TNM stage have been questioned (5, 6), and other scoring systems were established including Liver Cancer Study Group of Japan (7), Okabayashi et al. (8), and Nathan et al. (9). Besides, all these staging systems were based solely on variables limited to features of the primary tumor, and there remains a poor ability to predict prognosis for post-operative recurrence ICC patients, while the post-recurrence survival (PRS) of ICC patients is highly heterogeneous, and substantially impacted by features of recurrence and treatment modalities for recurrence (10–12). No studies before had specifically focused on the long-term outcome for post-operative recurrence ICC patients. Thus, it is imperative to develop a more precise prognostic scoring system in the management of patients with recurrent ICC in routine clinical practice.

Herein, we aimed to establish a prognostic nomogram specifically for post-operative recurrence ICC patients, and conduct a direct comparison with currently available staging systems.

## Materials and Methods

This study was conducted according to the ethical guidelines of the 1975 Declaration of Helsinki. All patients gave written informed consent, and the Institutional Review Board and Human Ethics Committee of Sun Yat-sen University Cancer Center approved this study's protocol.

### Study Population

We enrolled 237 patients with recurrent ICC after initial curative resection at Sun Yat-sen University Cancer Center between 2000 and 2016. Inclusion criteria: (1) primary tumor histopathologically confirmed as ICC; (2) confirmed recurrent ICC; (3) the margin status of the initial resection was microscopically negative (R0); (4) no history of other malignancies; and (5) no history of previous anticancer therapy before initial resection. Exclusion criteria were as follows: (1) hilar cholangiocarcinoma invading the liver; (2) mixed cholangiocarcinoma-hepatocellular carcinoma; (3) incomplete clinical data; and or (4) perioperative mortality.

### Diagnosis and Treatment

After a detailed history and a complete physical examination, patients' blood was tested for hepatitis B surface antigen, anti-hepatitis C virus (HCV) antibody, serum albumin, total bilirubin, alanine aminotransferase (ALT), γ-glutamyltransferase, α-fetoprotein, serum CA 19-9, and carcinoembryonic antigen (CEA). Other routine investigations included computed tomography (CT) or magnetic resonance imaging (MRI) of the abdomen and pelvis and radiography or CT of the chest at the discretion of the treating physician. Diagnoses of ICC after initial resection were based on histological evidence after resection.

### Hepatic Resection Procedure

Hepatic resection was performed using a previously described technique (13, 14). Partial hepatectomy was carried out according to tumor size, location, presence or absence of cirrhosis, and estimated volume of the future liver remnant. Anatomic resection was our preferred surgical method for multiple nodules in one segment or neighboring segments as described by Couinaud (15). A negative resection margin was defined as no residue under the microscope after resection at the margins nearest to the gross edge of the tumor. For ICC patients, lymph nodes were routinely evaluated using preoperative imaging, including CT and/or MRI of the abdomen and pelvis, and/or positron emission tomography (PET) if necessary. When lymph node metastasis, but not beyond the porta hepatis, was highly suspected preoperatively or intraoperatively, we dissected the hepatoduodenal ligament and retropancreatic and/or para-aortic lymph nodes as possible (16). However, the diagnostic accuracy, sensitivity, and specificity of imaging were insufficient for the accurate detection of lymph node metastasis (17). Thus, if retropancreatic and/or para-aortic lymph nodes metastasis was highly suspected intraoperatively, frozen-section pathological examinations were performed. Once retropancreatic and/or para-aortic lymph nodes metastasis was identified, curative resection was abandoned; as those lymph nodes involvement suggests metastatic disease and those groups of patients do not benefit from surgery (18).

Histopathologic study of the resected specimen was carried out independently by three pathologists; discrepancies were resolved via discussion and consensus. Pathologic features of the primary tumor were documented, including tumor number, size, capsule, site, surgical margin, vascular invasion, lymph node metastasis, cirrhosis, and tumor cell differentiation.

### Follow Up

Follow up was performed until June 30, 2019. After surgery, all patients were regularly followed, and prospectively monitored for recurrence with serum tumor marker and CT or MRI of the abdomen and a chest radiograph every 3–4 months up to 2 years and then every 6 months until year 5, followed by annual screening. Endoscopy, radionuclide bone scan, and PET were obtained as clinically indicated. ICC recurrence/metastasis was defined as the appearance of a newly detected tumor confirmed on two radiologic images, with or without elevation of serum tumor markers (10). The PRS was calculated from the date of the detection of the first recurrence to the date of death or the date of the last follow-up. Information regarding the tumor number, tumor size, tumor location, treatment for the recurrence, as well as the time from the date of initial operation to the development of recurrent disease was recorded. Sites of recurrence were categorized as intrahepatic, extrahepatic, or both.

### Treatment of Recurrence

The choice of treatment for recurrence was determined from the characteristics of the recurrent tumor, patient preference, and discussion among our multidisciplinary team (MDT) (19–21). Treatment with curative intent, including surgical re-resection and ablation, was performed in patients with solitary intrahepatic recurrence without a portal vein tumor thrombus, and in patients with an extrahepatic recurrence for which curative treatment was feasible (20–22). Other treatments were individualized for patients with advanced recurrent disease and included transarterial chemoembolization (TACE), chemotherapy and radiotherapy, depending on disease extent and the patient's performance status. Patients who were unable or refused to receive the aforementioned treatments were managed by the best supportive care.

### Statistical Analysis

Statistical analyses to identify risk factors were performed using SPSS 24.0 (SPSS, Chicago, IL) and R, version 3.5.3, software packages (http://www.r-project.org/). Continuous variables were compared using the *t-*test or Mann-Whitney *U-*test, and categorical variables were compared using the χ^2^ test or Fisher's exact test. Survival curves were constructed using the Kaplan-Meier method and compared with the log-rank test. Variables with a *P*-value (log-rank) <0.10 in univariate analysis were subjected to multivariate analysis using stepwise selection in a Cox regression model. Statistically significant factors (*P* < 0.05) from the multivariate analysis were entered into the nomogram. The optimal cutoff point for the total points of the nomogram was determined using the X-tile program. Model performance was evaluated through discrimination indicated by the Harrell C-index, and by plotting the Kaplan-Meier curves of the quartiles of predictions, and the performance was further illustrated by drawing calibration plots using a bootstrapped sample. Model validation was performed using bootstrap resampling to quantify the overfitting of our modeling strategy and predict the future performance of the model (23). The evaluation of the nomogram was performed through discrimination shown by the Harrell C-index and a calibration plot with a bootstrapped sample (16).

## Results

A total of 237 patients which were randomly divided into training (*n* = 178) and validation cohorts (*n* = 59) in the ratio of 3:1, were enrolled in this study. The clinical and pathological characteristics of ICC patients in the training and validation cohorts are summarized in Table 1. For the training cohort, 29 of 56 (51.8%) Cirrhotic patients underwent anatomical liver resection, while 74 of 122 (60.7%) non-Cirrhotic patients with anatomical liver resection. As for the validation cohort, 4 of 9 (44.4%) Cirrhotic patients underwent anatomical liver resection, while 33 of 50 (66.0%) non-Cirrhotic patients with anatomical liver resection. In the whole study, there are multiple lesions included tumors with satellites (*n* = 31; 13.1%) and tumors with contralateral lesions (*n* = 6; 2.5%) having resection. The median follow-up time was 22.8 months (range, 1.2–110.8 months) with 79 (44.4%) patient deaths, and 20.9 months (range, 1.8–89.2 months) with 25 (42.4%) patient deaths for the training and validation cohorts, respectively. The median PRS was 6.9 months (range, 0–78.0 months) and 7.3 months (range, 0–82.6 months) for the training and validation cohorts, respectively. The 1 and 3 years PRS rates were 37.6 and 8.4% in the training cohort, and 40.7 and 11.9% in the validation cohort, respectively.

**Table 1:** Demographics and clinicopathologic characteristics of patients.

**Variables**	**Training cohort (*****n*** **= 178)**		**Validation cohort (*****n*** **= 59)**	
	**No. of patients**	**%**	**No. of patients**	**%**
**Characteristics at initial liver resection**				
Age (y)				
≤60	120	67.4	36	61.0
>60	58	32.6	23	39.0
Sex				
Male	119	66.9	44	74.6
Female	59	33.1	15	25.4
History of hepatitis				
Yes	84	47.2	26	44.1
No	94	52.8	33	55.9
Alpha-fetoprotein (ng/mL)				
≤25	159	89.3	50	84.7
>25	19	10.7	9	15.3
Carbohydrate antigen 19–9 (U/mL)				
≤200	129	72.5	42	71.2
>200	49	27.5	17	28.8
Platelet count (×10^9^/L)				
≤100	6	3.4	0	0
>100	172	96.6	59	100.0
Liver function				
Alanine aminotransferase (U/L)				
≤40	131	73.6	49	83.1
>40	47	26.4	10	16.9
Prothrombin time (s)				
≤13.5	163	91.6	51	86.4
>13.5	15	8.4	8	13.6
Serum albumin (g/L)				
≤35	11	6.2	7	11.9
>35	167	93.8	52	88.1
Total bilirubin (μmol/L)				
≤20.5	155	87.1	52	88.1
>20.5	23	12.9	7	11.9
Liver cirrhosis				
Present	56	31.5	9	15.3
Absent	122	68.5	50	84.7
ALBI grade				
1	149	83.7	48	81.4
2	29	16.3	11	18.6
Tumor burden				
Greatest tumor size (cm)				
≤5	82	46.1	23	39.0
>5	96	53.9	36	61.0
Tumor number				
Single	156	87.6	44	74.6
Multiple	22	12.4	15	25.4
Macroscopic vascular invasion				
Present	38	21.3	7	11.9
Absent	140	78.7	52	88.1
Lymph node metastasis^a^				
N0	43	24.2	7	11.9
N1	28	15.7	11	18.6
NX	107	60.1	41	69.5
Capsule				
Incomplete	155	87.1	52	88.1
Complete	23	12.9	7	11.9
Anatomic resection				
Yes	103	57.9	37	62.7
No	75	42.1	22	37.3
Surgical margin (cm)^b^				
≤1	70	39.3	30	50.8
>1	108	60.7	29	49.2
Blood loss (ml)				
≤400	138	77.5	44	74.6
>400	40	22.5	15	25.4
Tumor differentiation				
Well	2	1.1	1	1.7
Moderate	123	69.1	43	72.9
Poor	53	29.8	15	25.4
Adjuvant therapy				
Yes	20	11.2	10	16.9
No	158	88.8	49	83.1
Characteristics at recurrence				
Time to recurrence (y)				
>2	11	6.2	1	1.7
1-2	42	23.6	8	13.6
≤1	125	70.2	50	84.7
Number at recurrence				
Single	83	46.6	22	37.3
Multiple	95	53.4	37	62.7
Size of recurrence (cm)				
≤5	153	86.0	57	96.6
>5	25	14.0	2	3.4
Site of recurrence				
Intrahepatic	104	58.4	32	54.2
Extrahepatic	17	9.6	9	15.3
Intra- and extrahepatic	57	32.0	18	30.5
CA19-9 at recurrence (U/mL)				
≤200	132	74.2	47	79.7
>200	46	25.8	12	20.3
ALBI grade at recurrence				
1	121	68.0	42	71.2
2	55	30.9	16	27.1
3	2	1.1	1	1.7
Treatment allocation^c^				
Supportive care	46	25.8	14	23.7
Non-radical treatment	64	36.0	20	33.9
Radical treatment	68	38.2	25	42.4

*HBV, hepatitis B virus; HCV, hepatitis C virus; ALBI, albumin-bilirubin*.

a *Lymph node metastasis: N0, negative; N1, positive; NX, lymph nodes not harvested*.

b *Surgical margin: the shortest measured distance from the edge of the tumor to the plane of liver transection*.

c *Non-radical treatment: TACE, systematic chemotherapy or radiotherapy; radical treatment: resection or ablation*.

### Independent Prognostic Factors in the Training Cohort

Using univariate analysis, possible correlations between PRS and 26 variables were evaluated for the 178 patients from the training cohort. Ten factors were related to PRS: serum CA19-9, platelet count, tumor size, tumor number, lymph node metastasis, time to recurrence, number at recurrence, serum CA19-9 at recurrence, albumin-bilirubin (ALBI) grade at recurrence, and treatment allocation (Table 2). Multivariate analysis demonstrated that serum CA 19-9, ALBI grade at recurrence, time from primary resection to recurrence, tumor number at recurrence, and treatment of recurrence were selected as five significant and independent risk factors for PRS.

**Table 2:** Cox proportional hazards regression model showing the association of variables with post-recurrence survival.

**Variables**	**Univariate analysis**		**Multivariate analysis**	
	**Hazard ratio (95% CI)**	***P*-Value**	**Hazard ratio (95% CI)**	***P*-Value**
Characteristics at initial liver resection				
Age, years (>60: ≤60)	1.079 (0.674–1.727)	0.751		
Sex (female: male)	1.042 (0.652–1.666)	0.862		
Hepatitis (yes: no)	1.141 (0.733–1.774)	0.559		
AFP, ng/mL (>25: ≤25)	1.502 (0.748–3.015)	0.252		
CA19-9, U/mL (>200: ≤200)	2.506 (1.527–4.114)	<0.001	2.318 (1.347–3.990)	0.002
PLT, ×10^9^/L (>100: ≤100)	0.327 (0.141–0.758)	0.009		
ALT, U/L (>40: ≤40)	1.349 (0.824–2.209)	0.234		
ALBI grade, (II: I)	1.611 (0.951–2.728)	0.076		
Liver cirrhosis (yes: no)	1.093 (0.690–1.730)	0.705		
Tumor size, cm (>5: ≤5)	2.093 (1.301–3.369)	0.002		
Tumor number (multiple: single)	2.016 (1.019–3.986)	0.044		
Lymph node metastasis^a^				
N0	1.0 [reference]	0.019 [reference]		
N1	2.117 (1.044–4.295)			
NX	0.920 (0.519–1.632)			
Capsule (complete: incomplete)	1.336 (0.721–2.475)	0.357		
Macroscopic vascular invasion (yes: no)	1.554 (0.879–2.747)	0.130		
Anatomic resection (yes: no)	0.996 (0.766–1.218)	0.769		
Surgical margin, cm (>1: ≤1)^b^	1.064 (0.880–1.286)	0.522		
Blood loss, ml (>400: ≤400)	1.323 (0.801–2.184)	0.275		
Tumor differentiation				
Well	1.0 [reference]	0.712 [reference]		
Moderate	2.093 (0.287–15.237)			
Poor	1.859 (0.249–13.889)			
Adjuvant therapy (yes: no)	1.027 (0.512–2.061)	0.940		
Characteristics at recurrence				
Time to recurrence, years				
≤1	1.0 [reference]	0.014 [reference]	1.0 [reference]	0.010
1–2	0.510 (0.296–0.881)		0.609 (0.333–1.115)	
>2	0.361 (0.130–1.003)		0.202 (0.067–0.245)	
Number at recurrence (multiple: single)	2.180 (1.377–3.453)	0.001	1.735 (1.062–2.834)	0.028
Size of recurrence, cm (>5: ≤5)	1.391 (0.780–2.482)	0.264		
Site of recurrence				
Intrahepatic	1.0 [reference]	0.058 [reference]		
Extrahepatic	1.038 (0.466–2.311)			
Intra- and extrahepatic	1.779 (1.099–2.881)			
CA19-9 at recurrence, U/mL (>200: ≤200)	2.643 (1.613–4.333)	<0.001		
ALBI grade at recurrence				
I	1.0 [reference]	<0.001 [reference]	1.0 [reference]	<0.001
II	2.420 (2.536–3.813)		2.549 (1.568–4.146)	
III	3.397 (0.816–14.140)		9.672 (1.896–49.334)	
Treatment allocation^c^				
Supportive care	1.0 [reference]	<0.001 [reference]	1.0 [reference]	<0.001
Non-radical treatment	0.609 (0.370–1.004)		0.663 (0.393–1.119)	
Radical treatment	0.144 (0.078–0.266)		0.135 (0.071–0.259)	

*95% CI, 95% confidence interval; AFP, alpha-fetoprotein; PLT, platelet; ALT, alanine aminotransferase; ALBI, albumin-bilirubin*.

a *Lymph node metastasis: N0, negative; N1, positive; NX, lymph nodes not harvested*.

b *Surgical margin: the shortest measured distance from the edge of the tumor to the plane of liver transection*.

c *Non-radical treatment: TACE, systematic chemotherapy or radiotherapy; radical treatment: resection or ablation*.

### Prognostic Nomogram for PRS

**Figure 1:**
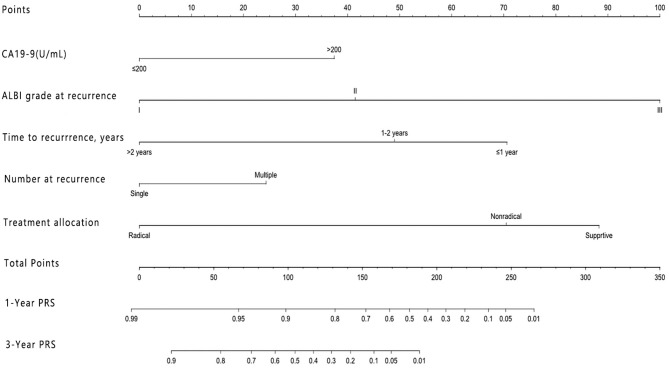
Nomogram for predicting the 1 and 3 years post-recurrence survival rates in patients with post-operative recurrence of intrahepatic cholangiocarcinoma.

The nomogram that included the aforementioned five identified independent risk factors for PRS is shown in Figure 1. The model illustrated that ALBI at the time of recurrence occupied the largest contribution to the PRS. By adding up each point and locating it on the total points scale, users could easily draw a straight line down to calculate the estimated 1 and 3 years PRS. Moreover, the use of X-tile v.3.6.1 software (Yale University, New Haven, CT) (24) the nomogram was able to accurately stratify patients into three prognostic subgroups with the scores of ≤99, 100–159, and >159. The 1-year PRS rates were 58.3, 36.1, and 17.5%, in these three subgroups of patients, respectively (*P* < 0.001).

### Validation of Predictive Accuracy of the Nomogram for PRS

**Figure 2:**
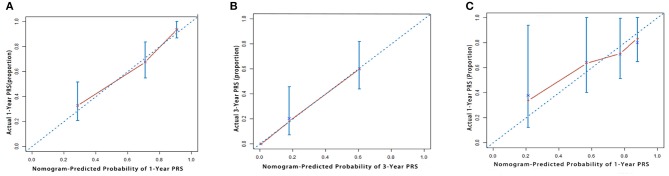
The calibration curve for predicting patient survival at **(A)** 1 year and **(B)** 3 years in the training cohort and at **(C)** 1 year in the validation cohort. Nomogram-predicted probability of overall survival is plotted on the *x*-axis; actual overall survival is plotted on the *y*-axis.

Our model showed better accuracy in predicting PRS in both training and validation cohorts compared with currently used staging systems. The C-index of the nomogram for PRS prediction was 0.791 (95% CI, 0.736–0.846), which was found to be significantly higher than the following systems: AJCC 8th edition 0.610 (95% CI, 0.542–0.678), Liver Cancer Study Group of Japan (LCSGJ) 0.613 (95% CI, 0.540–0.686), Nathan 0.582 (95% CI, 0.521–0.643), and Okabayashi 0.600 (95% CI, 0.537–0.663; *P* < 0.001 for all). In the validation cohort, our nomogram also displayed more accurate prediction than the abovementioned staging systems (C-index, 0.732 vs. 0.662, 0.642, 0.625, and 0.608, respectively; *P* < 0.001 for all). The calibration plot for the probability of 1 and 3 years PRS showed an optimal agreement between the prediction by nomogram, and actual observation both in training and validation cohorts (Figure 2).

### Discrimination Ability of the Prognostic Nomogram

**Figure 3:**
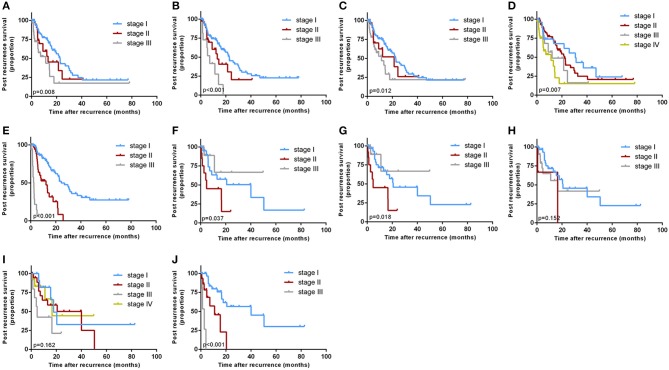
Kaplan-Meier survival curves categorized by different staging systems: the training cohort: [**(A)** American Joint Committee on Cancer (AJCC) 8th edition; **(B)** Nathan; **(C)** Okabayashi; **(D)** Liver Cancer Study Group of Japan (LCSGJ); **(E)** our nomogram]; the validation cohort: [**(F)** AJCC 8th edition; **(G)** Nathan; **(H)** Okabayashi; **(I)** LCSGJ; **(J)** our nomogram].

Kaplan-Meier curves were generated for all the staging systems, and the training and validation cohorts (Figure 3). Although the Kaplan-Meier curves showed different prognostic strata for all the staging systems in the training cohort (log-rank *P* < 0.05 in all cases), some overlapping of the survival curves was observed for all the other staging systems. In the internal validation cohort, the Kaplan-Meier curves showed clear prognostic strata for all staging systems (*P* < 0.05) except for Okabayashi and LCSGJ. The results reveal that the model was a useful predictor for the survival of patients with ICC in both training and validation cohorts.

## Discussion

The long-term outcome of patients after curative resection with ICC remains dismal due to a high incidence of recurrence (12). Thus, research that sheds light on the prognostic factors of post-recurrence survival of ICC patients desperately needs (25, 26). However, previous studies for ICC had focused almost exclusively on variables limited to characteristics of the primary tumor, and neglected the fact that the PRS of ICC patients were greatly impacted by features of recurrence and treatment modalities (9, 10, 16). By using a large patient cohort, our study firstly developed and validated a novel nomogram with more precise clinical utility for ICC patients compared with currently used staging systems.

As for the management of ICC patients, multiple algorithms had been proposed, such as AJCC TNM classification, LCSGJ (27), Okabayashi et al. (8), and Nathan et al. (9) staging systems. Although these staging systems enhance our overall understanding of tumor extension, they provide limited information about ICC patient-specific prognosis, due to the high heterogeneity ICC patients (28). The AJCC TNM stage is criticized as based solely on the anatomic extent of the ICC regardless of clinical factors or tumor biology (28). For the LCSGJ staging system, although it performed well in terms of prognosis stratification, it has not been widely validated or performed in countries other than Japan (9). The Okabayashi staging system has been criticized for the small number of patients as well as its poor prognostic applicability (29). Moreover, no studies before had specifically focused on the long-term outcome for post-operative recurrence ICC patients. Our study firstly highlighted the pivotal role of tumor characteristics at recurrence and treatment allocations in the PRS of ICC patients. The model we developed provided a precise, simplified but personalized approach in clinical practice to stratify ICC patients into different risk groups.

Guidelines for the management of recurrent ICC remain controversial and poorly defined. In the routine clinical practice, the therapeutic strategy for recurrence was evaluated based on tumor location, number of tumors, general patient condition, and liver function (20–22); however, the final choice was influenced by patient factors, including the preference for less invasive treatment and financial considerations decided by our MDT. The treatment allocation for recurrent ICC is recognized as a significant factor in this study. Radical treatments after recurrence, such as re-resection and ablation, exhibited long-term PRS compared to non-radical treatment for ICC (13.6 vs. 5.3 months), which was consistent with the previous study (19). The capability of radical treatments for recurrent ICC in offering a better chance to eradicate micrometastases generated from the tumor may explain to it (19, 30).

A cut-off of 1 year after resection has been used in previous studies to distinguish the early and late recurrence (31, 32). Patients relapsed within 1 year after initial resection may represent more aggressive tumor biology. In this study, time to recurrence, especially within a year after resection, had a significant impact on PRS, which was consistent with previous studies. Moreover, our model also included serum CA19-9 level at the time of initial resection, which has not been included as a variable in the currently used staging systems. The result of our study showed that the CA19-9 levels before the initial surgery and at recurrence were both recognized as significant factors in univariate analysis, while only the CA19-9 before initial surgery remained as a significant and independent predictor in multivariate analysis. Meanwhile, due to the more frequent surveillance after initial surgery, the size of tumor at recurrence (2.54 ± 2.21 cm) in our study was distinctly smaller than the primary tumor (5.93 ± 2.59 cm), and 26 (11.0%) patients suffered only extrahepatic recurrence, which may partly explain that why CA19-9 at recurrence was not related to PRS.

There are several limitations to this study. First, due to the retrospective nature of our study; second, the nomogram was established based on data obtained from a single institution; thus, deviation from this clinical design was unavoidable. To address this limitation, we enrolled in a large cohort consisting of 237 patients. Furthermore, consideration should be given to a prospective multicenter study to validate our findings.

In conclusion, we constructed a novel nomogram that objectively and precisely predicted the outcomes of patients with post-operative recurrence of ICC.

## Data Availability Statement

All datasets generated for this study are included in the article/supplementary material.

## Ethics Statement

This study was conducted according to the ethical guidelines of the 1975 declaration of Helsinki. The analysis of patient data was approved by the Institutional Review Board and Human Ethics Committee of Sun Yat-sen University Cancer Center. Informed consent was obtained from all patients included in this study.

## Author Contributions

K-LX, L-HL, XH, C-BH, Y-DS, R-PG, and S-PL: study concept and design and critical revision of the manuscript. K-LX, L-HL, and XH: drafting of the manuscript. C-BH and Y-DS: acquisition of data, analysis, and interpretation of data. K-LX, L-HL, C-BH, and Y-DS: statistical analysis. R-PG and S-PL study supervision. All authors read and approved the final manuscript.

### Conflict of Interest

The authors declare that the research was conducted in the absence of any commercial or financial relationships that could be construed as a potential conflict of interest.
